# Feasibility, acceptability, and sustainability of Project ECHO to expand capacity for pediatricians in Vietnam

**DOI:** 10.1186/s12913-021-07311-5

**Published:** 2021-12-09

**Authors:** Le Hong Nhung, Vu Duy Kien, Nguyen Phuong Lan, Pham Viet Cuong, Pham Quoc Thanh, Tran Minh Dien

**Affiliations:** 1 Vietnam National Children’s Hospital, No. 18/879 La Thanh Street, Hanoi, Vietnam; 2 OnCare Medical Technology Company Limited, No. 77/508 Lang Street, Hanoi, Vietnam; 3 Hanoi University of Public Health, No. 1A Duc Thang Road, North Tu Liem, Hanoi, Vietnam

**Keywords:** Feasibility, Acceptability, Sustainability, Project ECHO, Pediatrician, Vietnam

## Abstract

**Background:**

The Project Extension for Community Healthcare Outcomes (ECHO) model is considered a platform for academic medical centers to expand their healthcare workforce capacity to medically underserved populations. It has been known as an effective solution of continuing medical education (CME) for healthcare workers that used a hub-and-spoke model to leverage knowledge from specialists to primary healthcare providers in different regions. In this study, we aim to explore the views of healthcare providers and hospital leaders regarding the feasibility, acceptability, and sustainability of Project ECHO for pediatricians.

**Methods:**

This qualitative study was conducted at the Vietnam National Children’s Hospital and its satellite hospitals from July to December 2020. We conducted 39 in-depth interviews with hospital managers and healthcare providers who participated in online Project ECHO courses. A thematic analysis approach was performed to extract the qualitative data from in-depth interviews.

**Results:**

Project ECHO shows high feasibility when healthcare providers find motivated to improve their professional knowledge. Besides, they realized the advantages of saving time and money with online training. Although the courses had been covered fully by the Ministry of Health’s fund, the participants said they could pay fees or be supported by the hospital’s fund. In particular, the expectation of attaining the CME-credited certificates after completing the course also contributes to the sustainability of the program. Project ECHO’s online courses should be improved if the session was better monitored with suitable time arrangements.

**Conclusions:**

Project ECHO model is highly feasible, acceptable, and sustainable as it brings great benefits to the healthcare providers, and is appropriate with the policy theme of continuing medical education of the Ministry of Health. We recommend that further studies should be conducted to assess the impact of the ECHO program, especially for patient and community outcomes.

## Background

The Project Extension for Community Healthcare Outcomes (ECHO) model is considered a platform for academic medical centers to expand their workforce capacity to medically underserved populations [1]. Project ECHO uses a hub-and-spoke model through videoconferencing sessions to connect specialists (“hub”) at the center with generalists (“spokes”) in different regions [2]. Project ECHO was developed at the University of New Mexico in 2003, focusing on treatment for hepatitis C virus (HCV) infection in underserved populations and prisons [3, 4]. It was shown that HCV infection could be effectively managed in centers that implemented Project ECHO [5]. Due to this success, Project ECHO has been replicated and deployed in many countries to address common and complex health issues, such as HIV, substance-use disorders, asthma, pain management, rheumatologic conditions, and other ailments [6–12].

Project ECHO is also an effective solution to the continuing medical education (CME) process for healthcare providers. Zhou et al. used Moore’s Evaluation Framework for CME to evaluate different studies; they concluded that.

Project ECHO was an effective and cost-saving model to increase healthcare workers’ knowledge [13]. In addition, Project ECHO provided patients with more access to health care in remote areas. In particular, they found that the health status of patients improved because of changes in the practice behavior of healthcare providers who participated in Project ECHO [13]. Several other studies also indicated that health workers improved knowledge and professional competency via self-assessment of competency scale after taking online courses of Project ECHO [10–12, 14–18], and those things could improve the professional satisfaction at clinical practice. With changes in disease patterns and technological innovations in medicine, the CME process is necessary for healthcare worker’s professional competency development. Furthermore, in the circumstance of the current COVID-19 pandemic, the Project ECHO can be regarded as an option to help maintain CME/CPD for healthcare providers [19].

Several studies have evaluated the feasibility and acceptability of Project ECHO implementation in some countries [6, 20]. However, no studies have yet investigated the feasibility, acceptability, and sustainability of Project ECHO for pediatricians. Although Hostutler et al. conducted a study on pediatricians, this study only looked at improving physicians’ practice behavior after participating in Project ECHO [15]. In Vietnam, the tele-health project adapted Project ECHO was conducted to build HIV healthcare worker capacity [7]. This project was evaluated as successfully implemented and scaled up to improve healthcare worker capacity in HIV patient care. In 2019, Project ECHO for pediatricians started rolling out in Vietnam, and its online courses have been organized from the beginning of 2020 up to the time we conducted this study. This stage can be considered a start-up phase of the project, so it is necessary to have an assessment to implement this project more effectively. Thus, we conducted this study to understand the views of participants and hospital leaders about the feasibility, acceptability, and sustainability of the Project ECHO for pediatricians. In addition, we would also like to find out recommendations to make the Project ECHO better in the future.

## Methods

### Study setting

The study was conducted at the Vietnam National Children’s Hospital and its satellite hospitals. The study proposal was approved by research ethics committees at the Hanoi University of Public Health (261/2020/YTCCHD3) and the Vietnam National Children’s Hospital (883/BVNTW-VNCSKKTE). The Vietnam National Children’s Hospital is one of the leading pediatric hospitals in Vietnam, located in the North of Vietnam. The hospital plays the role of a direction center and provides a training system for satellite hospitals in pediatrics. The direction function of the hospital is represented in the form of updating documents and treatment guidelines for satellite hospitals on pediatrics, organizing training courses to update knowledge, and sending experts to support at the local hospitals in specific cases. The hospital accomplished certain achievements in providing professional support to the lower level, but professional support activities were not organized regularly due to limited resources, especially in remote areas.

The Project ECHO for pediatricians has been implemented since 2019 to improve the capacity of healthcare providers at provincial-level hospitals for the ECHO program conducted preparatory work in 2019, then the program officially recruited participants and organized online courses from the beginning of 2020. The structure of each session comprised two parts: the theoretical part (didactics) and the practical part (case study discussion). The theoretical part was based on pediatric knowledge basis and references from National Children’s Hospital’s education resource and partly from BMJ Best Practice e-learning. The practical part was based on actual cases prepared by the participants and facilitated by the course assistants. The process of organizing a training module included pre-course training, online learning, and offline self-study with resources of BMJ Best Practice. The content of the pre-course training included getting acquainted with how to operate the online system, how to access the BMJ Best Practice and learn about the course regulations. The online course included 8 to 10 sessions depending on the specific course, in which each session would last about 2 h with 1 h for theoretical presentation and the remaining 1 h for discussing a case. A typical online course was conducted in two afternoons per week for 3-5 weeks. The online learning process used the internet-based Zoom Video platform, in which the expert team (hub) sat at Vietnam National Children’s Hospital and participants (spoke) sat at satellite hospitals. The offline learning part was that students would be assigned lessons for self-study based on the relevant content in the BMJ Best Practice resource. The participants were assessed after the end of the course. If participants achieved from 60/100 points or more, and attended at least 80% of sessions, they would be considered achieved and granted a course completion certificate. In this study, we only focused on participants who participated in the course from January 2020 to June 2020, including courses on neonatal resuscitation, congenital heart diseases, and respiratory diseases in children. The study site was Vietnam National Children’s Hospital and 18 satellite hospitals which sent participants to participate in the online course. The participants did not have to pay for the courses during this time of study.

### Study design and sampling

**Fig. 1:**
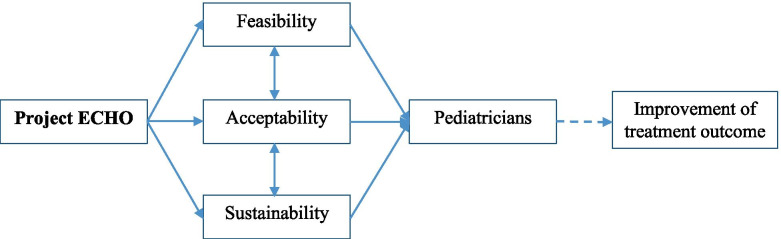
Conceptual framework for the feasibility, acceptability and sustainability of Project ECHO for pediatricians

We conducted the study with a qualitative research approach using thematic analysis to investigate the feasibility, acceptability, and sustainability of Project ECHO. The conceptual framework of the feasibility, acceptability and sustainability of Project ECHO for pediatricians, adjusted from Proctor et al.’s implementation research framework, was shown in Fig. 1 [21]. Besides, we evaluated the feasibility, acceptability and sustainability of Project ECHO based on the views of the participants [6, 10]. In addition, our previous study results showed that pediatricians’ clinical knowledge is significantly increased after the course [22]. Our findings are consistent with those from other settings, and again show the potential of the ECHO model in improving knowledge for medical staff at different levels [4, 13, 14, 23–26]. Some studies of Project ECHO implementation showed that learning goals were achieved through the ECHO training model that reduced feelings of professional isolation, increased providers’ confidence to manage any aspect of patient care [14, 16, 27], tightened clinical network through learning loops, broadened community of medical practice [10, 12, 28] and achieved CME [4, 29–34].

Qualitative data was collected through in-depth interviews with healthcare providers and hospital managers. The study was conducted between July and December 2020 with a total of 39 in-depth interviews. We have selected informants who are the system leaders and managers of the ECHO project at Vietnam National Children’s Hospital. At 18 satellite hospitals, we selected informants as participants of at least one online course from the project ECHO and hospital leaders who manage CME/CPD program. In each satellite hospital, we used the snowball technique to identify key informants with comprehensive experience about the project ECHO.

### Study tools and data collection

An interview guide was developed to assist with the in-depth interview process. The interview guide was written in Vietnamese, including general information collection of study participants (e.g., age group, sex, work experience in years and professional role) and content to learn about feasibility, acceptability, and sustainability of the Project ECHO for pediatricians. The interview guide also included content to learn about the training situation and understand the policy document related to pediatrician training. In addition, the interview guide included some questions regarding the informant’s recommendations for change to make the ECHO project better. Our interviews were conducted in meeting rooms at satellite hospitals. At the Vietnam National Children’s Hospital, we conducted interviews at the offices of the informants. All our interviews were held for 45 min to 1 h. LNH conducted all interviews with the assistance of a research assistant. At each satellite hospital, we stopped collecting information when the saturation of information was reached.

### Data management and analysis

The interview audiotapes were transcribed verbatim by a research assistant. The transcripts were reviewed and corrected by LHN and VDK. All transcripts were then translated into English and shared among co-authors for review and improvement. LHN and VDK discussed and finalized the transcripts. Data were entered into the OpenCode version 4.03 software [35]. We reviewed the verbatim interview transcripts several times to develop codes to capture the main content for each participant’s responses. The codes were grouped into different themes, reflecting the key concepts of the study, which included feasibility, acceptability, sustainability, and changes for the improvement of the ECHO program. The final thematic categories were reviewed and met the consensus by all authors.

## Results

### Sociodemographic characteristics

A total of 39 participants were enrolled in the study. Table 1 presents the participants’ general characteristics (e.g., age, sex, work experience, and professional role). The participants ranged between 25 and 59 years old, of whom the majority were between 25 and 39. Most participants were female (24/39), and most of them had work experience of less than 10 years (22/39). The majority of participants were pediatricians who provided consultancy and management for patients (25/39) and in the ECHO project, they were called participants. The rest of the participants had the professional role of hospital management (14/39), and in the ECHO project, they were known as the ECHO lead at spokes (14/39).

**Table 1:** The sociodemographic characteristics of the participants

Characteristic	Frequency
Age group (years)	
25–29	12
30–39	22
40–49	3
50–59	2
Sex	
Male	15
Female	24
Work experience (years)	
< 5	12
5–9	10
10–14	11
15–19	4
> 20	2
Professional role	
Providing consultation and treatment (ECHO participants)	25
Hospital manager	14
Total	39

### Unawareness of available policies, but high motivation to the professional knowledge

The policy on continuing medical education (CME) for health workers from the Ministry of Health (MOH) has been clearly stated, but the participants were unaware of this issue. Although unclear about the CME policy, most participants believed that they needed to continue studying to update knowledge and improve their professional skills. The participants were relatively passive in seeking and participating in the course, and most of them took the course because they were informed or appointed by the hospital leadership.

*"Actually, I don't understand the legal basis for continuing medical education. Maybe these things can be better understood from the hospital's leadership. The participation in the course depends on the approval of the clinical leadership." (physician - 10 years of experience)*

*“We don't know about the policy related to CME. When there is a bulletin about the course, we will be informed by the hospital leadership; if it is appropriate, we will ask our clinical manager to attend." (physician - 4 years of experience)*

Hospital leaders follow the policy of continuing medical education from the MOH. Leadership seems to support this kind of training to improve knowledge and professional competency for their medical staff in order to scale up its reputation and attain the final goal of improved quality of service delivery performance.

*“Hospital leadership focus on training, which includes both in-hospital training and tele-training. These two forms of training are important because they help promote the quality of human resources for the hospital. Not only that, the participation in training is appropriate with the policy on the continuing medical education of the Ministry of Health.” (hospital leader - 15 years of experience)*

*“The general policy of the hospital has been to encourage staff in training program attendance to update their knowledge.” (clinical manager - 15 years of experience)*

### Save money and time, but still ensure work at the hospital

The ECHO program created a lot of favorable conditions for participants. The participants said that they could save time, accommodation and travel costs while securing their assignments at the hospital. Moreover, the participants had the opportunity to learn from peer-to-peer interactions, and short didactic training from specialists in different locations at the same time via online learning.

*"When learning online, it is convenient for us because we can both work and update our technical knowledge (physician - 12 years of experience)*

*“Firstly, online learning in the ECHO program saves money. Next, the ECHO program attracts more trainees to participate. Especially, the training curriculum satisfies the needs of participants. Finally, the participants had the opportunity to discuss with each other and the experts to improve the quality of learning.” (physician - 5 years of experience)*

Most participants agreed that the courses from the ECHO program was very useful and met their expectations. Although the trainees were confused by the virtual training at first, they soon adapted to the new method of training after a few sessions. In particular, the participants found that the course was a good mix of theory and clinical case studies.

*“I found that the course provided by the ECHO program very useful and met my expectation. Thanks to the course, I was able to update my professional knowledge. I hope that there will be more courses like this in the future.” (physician - 3 years of experience)*

*“At first, when I approached the new way of learning, I was very confused, because I did not know how to connect into the online classroom and how to interact with. Fortunately, after being guided and experienced sometimes, I found it easy and convenient to use.” (physician -5 years of experience)*

### Class time and internet connection may affect participation

Some participants also felt that the time of the lesson should be adjusted appropriately. Participants suggested that the time for clinical case discussion should be increased. The total amount of time for each session should also be adapted, according to some, to help participants balance between working time and family assignments.

*"It is necessary to adjust the study time to assure that participants can attend fully. Particularly, the teachers should spend more time to discuss the clinical cases." (physician - 10 years of experience)*

*"If the session time prolonged so much, it would be a barrier for participants because they are busy with working and housework." (physician - 5 years of experience)*

Most participants thought that being connected to the online classroom was convenient because different devices could be used such as PC, tablet and smartphone could be used. However, some experienced IT issues may have affected their study.

*“The biggest problem for us is IT connection. Sometimes due to low-quality network, my connection to the classroom was interrupted. Thus, it will affect the acquisition of knowledge in the session.” (physician - 3 years of experience)*

### High acceptance from both hospital leaders and participants

Most hospital leaders recognized the fidelity and appropriateness of the ECHO program for professional competency building. The courses offered in the ECHO program would help reduce patient overloading in patient concentration in the central-level hospitals. In particular, in the complicated spread of the COVID-19 pandemic, distant training helps prevent the disease infection.

*“Project ECHO is a very practical model, appropriate with the Ministry of Health's policy on continuing medical education. Moreover, this model will urge to fill the gap in knowledge between the central and local physicians. Consequently, it might reduce the patient's overloading concentration in examination at the central-level hospital. We have more skills in detecting complicated cases quickly to transfer to the upper-level hospital”.” (hospital leader - 22 years of experience)*

*“Currently, the COVID-19 pandemic is complicated, so the implementation of the ECHO program is relevant to prevent the spread of the disease and update the knowledge of healthcare worker.” (physician - 5 years of experience).*

### The content of the course determines acceptability

Most of the participants highly appreciated the quality of the course. In particular, the participants all acknowledged that a large number of staff were trained through the ECHO training program. The participants shared that participating in the course helped them gain more confidence in their clinical practice. Furthermore, they stated that the program guided better practice in the pediatric department. In fact, there was a large gap in professional knowledge between medical staff at the central-level and the provincial-level, the provincial level and the district level. The ECHO learning model shortens the gap in professional knowledge between central-level experts and different level physicians.

*“Through the ECHO online training program, it is possible to train a large number of staff at the same time without having to travel.” (physician - 6 years of experience)*

*“Joining in the ECHO program helps us to gain more confidence in diagnosing, managing patients from complying strictly with the protocol shared by the expert panel. I used to examine similar cases in the past, but I failed in managing and treatment patient due to lack of evidence-based protocol, just counting on the experience” (physician - 4 years of experience)*

### Formation of a virtual learning community among the expert panel and peer-to-peer colleagues strengthen acceptability

Most participants found that they acquired knowledge a lot from the case study discussion. Through the cases being shared and discussed, participants got access to updated clinical protocols and expertise experiences from specialists in pediatrics and their peers at different hospitals. As a result, they built the community of “all teach all learn” through the exchange of expertise and best practice in patient management.

*“The course gave us an opportunity to exchange knowledge among our peers and mentors to deal with difficult cases from different hospitals. We can easily take a personal rapport with mentors and colleagues in case of asking for consultancy in some urgency and patient treatment” (physician - 12 years of experience)*

### The sustainability based on the content of the curriculum and expectations on CME achievement

The ECHO program is sustainable because the current implementation cost is fully covered by the government under the Ministry of Health. Most of the participants said that the curriculum content was the main reason for them to attend. However, some participants believed that if the course charges a fee, they would consider participating. The expensive tuition may be a barrier for participants to attend the course, so the payment should be dealt with the hospital fund”.

*"The tuition is not a big problem for me, I care about the program curriculum we will receive. Can the course provide us with the necessary knowledge or not? Does the course serve my specialty?" (physician - 15 years of experience) “I think training for medical staff at the hospital is compulsory. Currently, self-financed hospitals have funds for staff training. Therefore, if the payment is appropriate, the hospital can afford it.”*

In addition, the expectation of achieving the CME-credited certificate after completing the course also contributes to the sustainability of the ECHO program. Thus, CME attainment that assures the professional certificate of each individual would be a significant motivation for the participants to attend and satisfy the post-assessment of clinical knowledge and self-efficacy. Moreover, the quality of didactics also needs to be paid attention to carefully, leading to the high quality of the courses.

*“The program is highly sustainable because it improves professional knowledge and issues a certificate of continuous training according to the Ministry of Health regulations. If there is a certificate for continuous training, it will motivate participants to join the program.” (clinical manager - 10 years of experience)*

*“It is necessary to assure human resources for teaching. There should be organized expert panel recruitment with enough capacity to specialize at their faculties and comply with the training discipline besides possessing the pedagogical degree. Similarly, provincial-level physicians also need to be encouraged to study and critically participate in the course.” (clinical manager - 22 years of experience)*

### Changes to improve project ECHO

Because the courses are held online, it is essential to monitor the number of participants in the lessons. Some participants recommend that ECHO courses need better supervision of student participation. In addition, the choice of time for the study sessions is also important affecting student participation. The class time arrangements should be chosen in order to facilitate the participant’s working time and time for family care. In order to achieve improvement in the organization, besides monitoring participation from the host, it is necessary to call for the support in supervision from the spokes. Some participants also said that it was necessary to increase the time for case study discussion in each session and organize more diverse and frequent pediatric topics

*"The issue of course supervision can be a challenge because all activities happen online. So, it requires the technical team to have enough capability to control and support the participants from a distance since if participants do not attend the course fully, it can affect the quality of training." (physician - 5 years of experience)*

*“Besides monitoring the course from the hub, there is also a need for monitoring support at spokes. Monitoring the participant’s attendance at spokes will increase the efficacy of the course.” (clinical manager– 15 years of experience)*

*“I think that it should be increasing the time for case study discussion. Besides, the organization should host some more courses with diverse topics and more frequency.” (clinical manager -15 years of experience)*

## Discussion

This is the first study in Vietnam to investigate the feasibility, acceptability, and sustainability of Project ECHO to expand the capacity of pediatric healthcare providers throughout its system. The results of the study show that the program is feasible, acceptable to healthcare providers and hospital leaders, and likely sustainable for competency improvement in satellite hospitals. Project ECHO is in line with the policy of MOH on CME for healthcare providers and has notable advantages during the time of COVID-19 pandemic spreading with the governmental regulation on social distancing and lockdowns.

We found the ECHO project feasible because it met the needs of the improvement in medical staff’s capacity building and their job satisfaction at the workplace. Besides, healthcare providers must comply with the policy of MOH about participating in continuing education with at least 48-h within 2 years to maintain their work permit certificates. In fact, the continuous learning applied the ECHO model had improved the capacity of HIV healthcare workers in Vietnam a great deal that 90% of participants wanted to participate in the next courses [7]. Another study also indicated that the pediatric care providers improved their professional knowledge and skill after following Project ECHO training [15, 22]. Our findings are consistent with those from other settings [4, 13, 14, 24–26], and again show the potential of the ECHO model in improving knowledge for medical staff at different levels.

While most of the medical staff were unaware of the policy regarding CME, which was considered a matter of concern, the hospital leaders acknowledged well about it. Through the interviews done with hospital leaders, especially clinical managers, most of them expressed their strong points on the support for the course implementation from their hospital, including funds and human resources, which are being regarded as key elements to make the ECHO program feasible. We learned that the MOH should leverage this policy broadly throughout the medical system and collaborate with hospital leaders to disseminate this information to medical staff.

The fact is that there is an overcrowding concentration of patients at the central-level hospitals while there still exists the shortage of competent workforce at the satellite hospitals. So that, hospital leaders have a tendency to show strong desires in medical staff training. Most of the healthcare providers indicated that they were encouraged by hospital leaders to participate in the courses provided by Project ECHO for the professional knowledge improvement. Another qualitative study concluded that it would be a considerable barrier if the hospital leaders did not permit the medical staff to attend the ECHO course [36]. Therefore, the approval and support from the hospital leaders in this study have been vital in making Project ECHO feasible in Vietnam.

The convenience of taking online courses from Project ECHO was greatly appreciated by the participants. The way of learning using online tools allowed the participants to balance their time of working and training. Moreover, Project ECHO was regarded as an excellent solution to help these healthcare providers save time and money in accommodations and traveling while they could fulfill their assignments at their workplaces. These advantages were mentioned in the previous studies about the ECHO model implementation [36, 37]. Besides, the ECHO model also provided an opportunity for the participants to discuss with peers among different spokes and to be mentored by an expert panel through case-based learning. The presentations counted on the evidence-based approaches may create more inspiration for participants to pursue the following courses.

Although the advantages of Project ECHO have been apparent, some obstacles happened that prevented the implementation of the ECHO program. Firstly, the timing of the session could affect the physician’s engagement in the course. If the ECHO sessions were held during working hours, the participants would find it hard to fulfill their assignments. Similarly, if the ECHO session was held after working hours, the participants might not join because of family responsibilities. This issue could be solved by the commitment of the hospital leaders to arrange an appropriate time for their staff to engage in the course during working time. Secondly, it was the internet connection problem that affected the participant to join the class. Thus, it is necessary for satellite hospitals to set up an online network that is well-connected enough to satisfy the demand of device preparation for the ECHO course.

The success of an ECHO program critically depended on the acceptability from both the healthcare providers and hospital leaders. Before this study, Damian et al. evaluated the level of satisfaction through the survey with participants as a tool to measure the program acceptability [20]. However, the satisfaction was not persuasive enough to reflect the acceptability. Recently, De Witt Jansen et al. used in-depth interviews to assess the acceptability of the ECHO program for dementia. The learners shared that they accepted the program because they accessed the expert knowledge and skills that helped them in clinical practice [17]. Thus, in this study, we explored the acceptability focused not only on healthcare providers’ views like some previous studies but also on hospital leaders’. The result showed that the hospital leaders were fully aware of the expertise of the healthcare workforce associated with the development of healthcare services they supply and the improvement of patient outcomes they manage. Therefore, they always encouraged their medical staff to join the continuous training and even committed to supporting the tuition fee from the hospital fund if needed. For the healthcare providers, their acceptance was based on the quality of the course and the model of peer-to-peer learning and expert panel mentorship. In general, the acceptability from the hospital leaders and healthcare providers at a high level could allow the expansion of the ECHO model in Vietnam.

The sustainability for the implementation of Project ECHO was considered an important element to maintain and replicate the program. In fact, Project ECHO for pediatricians has been sponsored by governmental funds that charged free for participants following the “Satellite Project” [38]. Thus, as the Project ECHO has to run by its resource in the future, it is essential to examine the factors that impact the program’s sustainability. Although previous studies have mentioned the feasibility and acceptability of Project ECHO [6, 14, 15, 20], few researchers have studied the sustainability of the program [7, 39]. To ensure the sustainability of Project ECHO, the financial issue is crucial because the project has to invest in human resources, devices and the online network [39, 40]. We learned that the hospital leaders supported the ECHO program by allowing their staff to join, ensuring sustainability. Moreover, the healthcare providers stated that they would be willing to pay tuition fees for the courses if the curriculum contents satisfy their expectations. The fact was that the acceptability and feasibility brought from the learner’s expectation in the course also contributed considerably to the sustainability. Besides, the sustainability of Project ECHO is counted on its consistency with the MOH’s policy on CME.

Several studies showed the effectiveness of using the ECHO model in the management of various diseases with improved clinical outcomes [14, 41–44]. However, there have been few studies about Project ECHO that applied for pediatrics [15]. We implemented the Project ECHO for pediatricians to expand the expertise knowledge in some faculties related to pediatrics, including Neonatology, Pediatric respiratory diseases, Pediatric Cardiology, to the distant area throughout Vietnam [22]. Thanks to the IT innovation recently in Vietnam, especially in the condition of Covid-19 pandemic spreading, the distance learning has become more urgent than ever before to satisfy the social demands of distancing that have attracted a large number of participants to join. Therefore, it is necessary for the organization implemented Project ECHO to pay attention to the expectation of learners on the quality of education and relevant methodology that will meet their demand of long-life learning.

To sustain and replicate the Project ECHO-Pediatrics to Vietnam healthcare system, we propose the MOH continue funding for this activity and integrate this model into the “National tele-health project” that will be implemented widely throughout Vietnam from the central to the community level starting in 2020 [45–47]. Another indication for sustainability and replication of Project ECHO-Pediatrics would have the stable funding from pharmaceutical companies, hospital’s workforce development funding, and VNCH co-funding mechanism.

### Study limitation

Although the qualitative design helped us explore in-depth the opinions of healthcare providers and hospital leaders about the feasibility, acceptability, and sustainability of Project ECHO for pediatricians, our study has some limitations. First, we used snowball sampling to identify informants. Therefore, we may have been referred to people who were more interested in Project ECHO, causing the results to not fully reflect participants’ views. In addition, we only conducted in-depth interviews at some of the satellite hospitals. Thus, there might be some participants with different views located at the hospitals that were not examined. Moreover, due to the nature of qualitative studies and their basis on in-depth interviews, the generalization of the results should be made with caution.

## Conclusions

Project ECHO model is highly feasible, acceptable and sustainable as it brings great benefits to the healthcare providers, and is consistent with the MOH’s policy for CME. However, it is necessary to raise awareness of the policies and regulations related to CME for healthcare providers, thereby creating an incentive for individuals to take part in available courses. In the context of the COVID-19 pandemic, the delivery of Project ECHO courses offers significant practical advantages. It allows for social distancing while ensuring that participants’ learning goals are met. Therefore, Project ECHO model needs to be sustained and replicated. For further development, the organization board from the hub should pay attention to the expert panel’s quality, the curriculum content, class time arrangement, and quality assessment and supervision. We believe that there will be more studies on the improvements in medical knowledge and the professional competency of healthcare providers taking ECHO courses, as well as further assessments of improvements in patient outcomes.

## Data Availability

The datasets used and/or analysed during the current study are available from the corresponding author on reasonable request.

## Abbreviations

CME: Continuing Medical Education
ECHO: Extension for Community Healthcare Outcomes
HCV: Hepatitis C Virus
HIV: Human Immunodeficiency Virus
MOH: Ministry of Health
WHO: World Health Organization
